# Computational Screening for the Dipeptidyl Peptidase-IV Inhibitory Peptides from Putative Hemp Seed Hydrolyzed Peptidome as a Potential Antidiabetic Agent

**DOI:** 10.3390/ijms25115730

**Published:** 2024-05-24

**Authors:** Arisa Thongtak, Kulpariya Yutisayanuwat, Nathaphat Harnkit, Tipanart Noikaew, Pramote Chumnanpuen

**Affiliations:** 1 Mahidol Wittayanusorn School, 364 Salaya, Phuttamonthon District, Nakhon Pathom 73170, Thailand; arisa.thongtak@gmail.com (A.T.); eungkulpariya2235@gmail.com (K.Y.); 2 Medicinal Plant Research Institute, Department of Medical Sciences, Ministry of Public Health, Nonthaburi 11000, Thailand; dough.nathaphat@gmail.com; 3 Department of Biology and Health Science, Mahidol Wittayanusorn School, 364 Salaya, Phuttamonthon District, Nakhon Pathom 73170, Thailand; tipanart.noi@mwit.ac.th; 4 Department of Zoology, Faculty of Science, Kasetsart University, Bangkok 10900, Thailand; 5 Omics Center for Agriculture, Bioresources, Food and Health, Kasetsart University (OmiKU), Bangkok 10900, Thailand

**Keywords:** *Cannabis sativa*, bioinformatic, dipeptidyl peptidase-IV, diabetes

## Abstract

Dipeptidyl peptidase-IV (DPPIV) inhibitory peptides are a class of antihyperglycemic drugs used in the treatment of type 2 diabetes mellitus, a metabolic disorder resulting from reduced levels of the incretin hormone GLP-1. Given that DPPIV degrades incretin, a key regulator of blood sugar levels, various antidiabetic medications that inhibit DPPIV, such as vildagliptin, sitagliptin, and linagliptin, are employed. However, the potential side effects of these drugs remain a matter of debate. Therefore, we aimed to investigate food-derived peptides from *Cannabis sativa* (hemp) seeds. Our developed bioinformatics pipeline was used to identify the putative hydrolyzed peptidome of three highly abundant proteins: albumin, edestin, and vicilin. These proteins were subjected to in silico digestion by different proteases (trypsin, chymotrypsin, and pepsin) and then screened for DPPIV inhibitory peptides using IDPPIV-SCM. To assess potential adverse effects, several prediction tools, namely, TOXINpred, AllerCatPro, and HemoPred, were employed to evaluate toxicity, allergenicity, and hemolytic effects, respectively. COPID was used to determine the amino acid composition. Molecular docking was performed using GalaxyPepDock and HPEPDOCK, 3D visualizations were conducted using the UCSF Chimera program, and MD simulations were carried out with AMBER20 MD software. Based on the predictive outcomes, FNVDTE from edestin and EAQPST from vicilin emerged as promising candidates for DPPIV inhibitors. We anticipate that our findings may pave the way for the development of alternative DPPIV inhibitors.

## 1. Introduction

Based on mortality rates in Thailand, diabetes stands out as a significant non-communicable disease. Presently, Thailand has over 4 million diabetes patients, with only 35.6 percent of them achieving their treatment targets. Type 2 diabetes (T2DM) is more prevalent than type 1 diabetes (T1DM), accounting for up to 90 percent of all diabetes cases [1]. It can arise from genetic factors or lifestyle elements like obesity, unhealthy diet, age, and smoking [2], leading many individuals to develop severe complications, including heart disease and diabetic ulcers, at a later stage [3].

Dipeptidyl peptidase-IV (DPPIV), belonging to the prolyl oligopeptidase family of serine proteases, removes dipeptides from the N-terminus of various substrates, including chemokines, neuropeptides, and peptide hormones. Consequently, increased activity and expression of DPPIV are significant risk factors for diabetes [4]. DPPIV speeds up the cleavage of the dipeptide at the amino end of incretins such as glucagon-like peptide-1 (GLP-1) and glucose-dependent insulinotropic polypeptide (GIP), reducing its half-life, which plays a role in regulating blood glucose homeostasis [5]. Some studies suggest that serum DPPIV activity is higher in diabetic patients compared to non-diabetic individuals [6]. DPPIV proteins highly expressed in diabetic patients [7] may be caused by chronic low-grade inflammation due to spontaneous glycation, causing cell signaling like NF-kB [8,9] which results in the induction of TNF-alpha, IL-1, and IL-6 [10], cytokines that regulate DPPIV messenger RNA [11]. DPPIV is expressed in cells such as intestinal epithelial cells [12], which accelerate the breakdown of incretin hormone groups including glucagon-like peptide-1 (GLP-1) and glucose-dependent insulinotropic polypeptide (GIP). Incretin hormones are highly associated with insulin secretion [13].

Currently, DPPIV inhibitors used in the treatment of diabetes can still lead to various adverse effects. These include allergic reactions to diabetes medication and disruptions to the digestive system resulting in diarrhea, infections, constipation, gastritis, and skin inflammation [14]. Rather than relying on chemical and synthetic drugs, peptide-based drugs derived from natural sources are increasingly used for treating various diseases due to their enhanced target specificity and cost-effectiveness [15,16]. Natural and synthetic peptides were considered in this project due to their various beneficial reports. Most DPPIV inhibitory peptides are derived from plant-based sources, which cost less compared to animal-based proteins [16]. Peptides from plants such as macadamia and jack bean have been reported to have DPPIV inhibitory peptide properties [17,18], which is one of the curative methods for diabetic patients. 

Hemp, or *Cannabis sativa*, belongs to the *Cannabis* genus of the Cannabaceae family. Hemp seed is gaining attention as an alternative plant protein source due to its high protein content and balanced amino acid profile, comprising 20–25% protein, which can vary based on environmental factors [19,20,21,22]. Edestin, a specific globulin protein, is the most abundant protein in hemp seed. Together with albumin, the second most prevalent protein, they make up 60–80% of the seed’s protein content, while vicilin is present in minimal amounts compared to other seed proteins [19,23,24]. The health benefits of hemp seed protein hydrolysate show promise. Numerous studies, using both computational and experimental approaches, have explored the potential advantages of these peptides and their derivatives [23,25,26]. Additionally, numerous in silico and in vitro studies have reported on the biological activities of hemp seed peptides. These activities include lipid-lowering, cholesterol-lowering [27], antioxidative [28,29], anti-melanogenic [30], anti-fatigue [31,32], antihypertensive [33,34], anti-inflammatory and immunomodulatory [35,36], hypoglycemic [37], antiviral [25], and anti-allergenic effects [38]. Even though previous research experimentally identified some candidates for functional hypoglycemic peptides from hemp seed hydrolysate, they were proposed to function through α-glucosidase inhibitory activity rather than DPPIV inhibition [37].

Traditional experimental approaches can precisely assess the DPPIV inhibitory activity of peptides, though these methods are time-consuming and expensive. Recently, machine learning (ML) has proven effective in predicting the properties of proteins and peptides using only their primary sequence, eliminating the need for prior knowledge or 3D structure information [39]. In general, ML-based predictors are employed for screening bioactive peptides and selecting candidates based on the preferred distribution patterns of amino acid compositions. Using these bioinformatics approaches, previous studies have shown that several antidiabetic peptides can be identified in soybean and black bean hydrolysates, potentially exhibiting properties beneficial for treating type 2 diabetes [40,41].

This study aimed to assess the potential and bioavailability of hemp seed peptides, with the intention of further development for functional food and diabetic treatment purposes. The objective of this study was to conduct virtual screening and predict DPPIV inhibitory peptides from the putative peptidome of hemp seeds using our developed bioinformatics pipeline. Both molecular docking and molecular dynamics simulations were employed for the in silico validation of inhibitor–enzyme interactions. The DPPIV inhibitory peptide candidates identified in this study could be further developed as an alternative treatment for type 2 diabetes.

## 2. Results and Discussion

### 2.1. Hemp Seed Putative DPPIV Inhibitory Peptide Screening Using a Bioinformatics Approach

**Figure 1:**
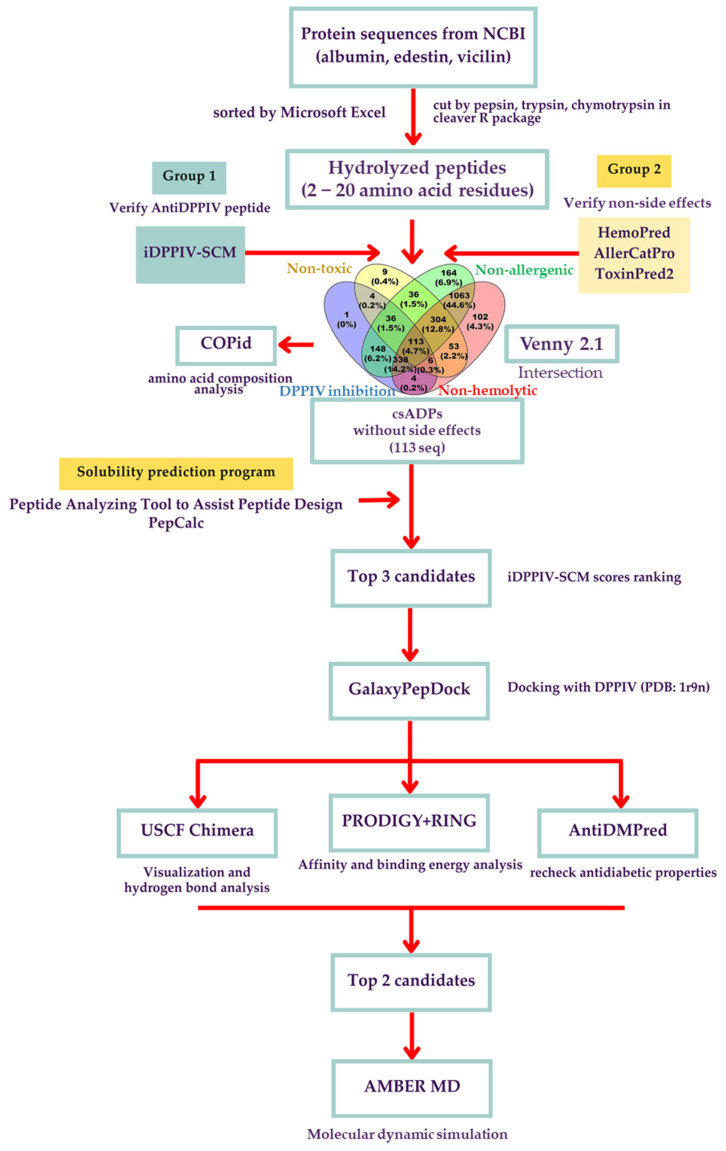
The workflow of the bioinformatic virtual screening for *Cannabis sativa* antidiabetic peptides (csADPs) with DPPIV inhibitory activity and the in silico analysis of DPPIV protease inhibition using molecular docking–molecular dynamics simulations.

**Figure 2:**
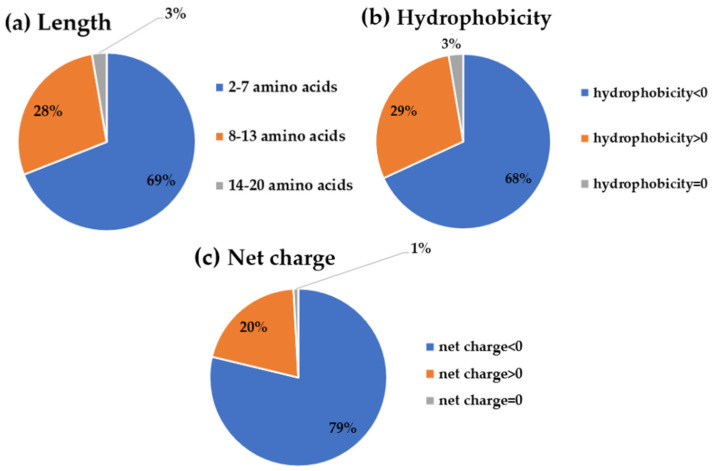
Percentage of the peptides’ properties regarding their (**a**) length, (**b**) hydrophobicity, and (**c**) net charge from 113 putative csADPs.

To obtain the most potential antidiabetic peptide candidates with the desired properties, the bioinformatics pipeline for computer-aided screening and validation was carefully designed, as illustrated in (Figure 1). Firstly, a dataset of the putative peptidome from all high-abundance hemp seed proteins (albumin, edestin, and vicilin) was generated by in silico enzymatic digestion, simulating the predicted cut results of specific protease enzymes on the original protein sources from *Cannabis sativa* subsp. *sativa*. Hemp seed protein is primarily composed of three types of proteins: 11S globulin (edestin), 2S albumin, and 7S vicilin-like protein, which account for approximately 60–80%, 13%, and 5% of the total seed protein, respectively [42,43]. Although the vicilin content is lower than that of edestin and albumin, it remains one of the top three most abundant proteins in hemp seeds. Previous research has consistently proposed vicilin-derived peptides for various bioactive functions, underscoring its significance despite its lower proportion [19,25,26,44]. In this study, three specific protease enzymes (pepsin, trypsin, and chymotrypsin) were selected to provide a more diverse hydrolysate peptidome dataset compared to our previous results with the putative trypsinized peptidome [25]. As suggested by previous research, only peptides containing 2 to 20 amino acids were selected for further study because this length has been reported to be the most effective for DPPIV inhibition [45] and was accessible for our program prediction list. From a total of 2393 putative hemp seed peptide sequences, peptides were predicted to be *Cannabis sativa* antidiabetic peptides (csADPs) with DPPIV inhibitory activity if their score exceeded 294 in iDPPIV-SCM. Moreover, only candidates without side effects, including non-allergenic, non-hemolytic, and non-toxic properties, were considered. The predicted evidence for allergenicity needed to show as ‘no evidence’ in AllerCatPro, results needed to be ‘non-hemolytic’ in HemoPred, and the probability needed to be lower than 0.6 in ToxinPred2, respectively. As a result, iDPPIV-SCM predicted 650 peptides, AllerCatPro predicted 2202 non-allergenic peptides, HemoPred predicted 1983 non-hemolytic peptides, and ToxinPred predicted 561 non-toxic peptides. From these four programs, only 113 peptide candidates met all criteria as potential antidiabetic peptides with DPP-IV inhibitory effects and without undesired side effects (Figure 1). In terms of peptide physicochemical properties, the majority of putative DPP-IV inhibitory peptides were 2 to 7 amino acids long (69%), hydrophilic (68%), and anionic (79%) (Figure 2).

In addition, to ensure the water solubility of our peptide candidates for further drug development, water-soluble properties were calculated using PepCalc and the Peptide Analyzing Tool to Assist Peptide Design. The results of the peptide screening session suggested that the desired csADPs with high water solubility and non-side effects were high in 2–7 amino acid peptides, like the typical DPPIV inhibitory peptides stated in the previous report [45,46].

**Figure 3:**
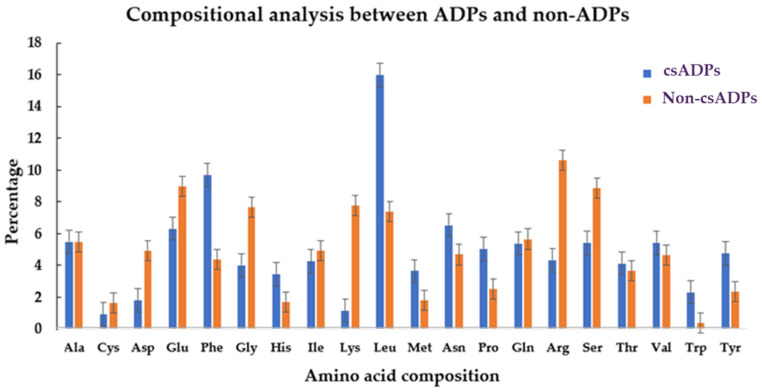
The compositional analysis represents the preferences between the significant csADPs and non-csADPs.

To also assess the different preferred amino acid distributions, a comparison of the amino acid composition between the putative csADPs and non-csADPs was analyzed using the COPid program, as shown in Figure 3. From a comparison of the amino acid composition of csADPs and non-csADPS by COPid, the amino acid residues with significantly higher percentages in csADPs were Leu, Phe, and Pro. Leu and Phe are hydrophobic amino acids that may play an important role in hydrophobic interactions at active sites. Similar to what has been suggested by previous research, DPP-IV inhibitory peptides usually contain hydrophobic amino acids (Leu, Pro, Trp, Met, and Val) [47]. However, other aromatic amino acids (Phe, Trp, and Tyr) and small, basic side-chain amino acids like His were also found to be the most enriched in the composition of csADPs, possibly contributing to their non-side-effect properties. For example, positively charged amino acids were proposed to be enriched in the non-hemolytic peptide group [48]. According to a previous report by Liu et al. (2019) [49], N-terminal hydrophobic residues influence hydrophobic binding interactions at the active site of DPPIV. However, our results differ slightly, with csADP candidates consisting of up to 68 percent hydrophilic amino acids. Moreover, they are likely to be anionic. The three top-ranked protein sources were from edestin- and vicilin-like proteins all hydrolyzed by pepsin. 

The top-ranked csADPs, which were water-soluble and had no side effects, were selected based on their predictive probability of iDPPIV-SCM. All 113 peptides from the common predicted peptides with water-soluble properties were sorted, and three peptides, including NVDTE, EAQPST, and FNVDTE, were among those with the highest scores (iDPPIV-SCM). These three potential antidiabetic peptide candidates from *Cannabis sativa* seed were named csADP1 (NVDTE), csADP2 (EAQPST), and csADP3 (FNVDTE) for further procedures (Table 1).

### 2.2. Protein–Peptide Docking Simulations

**Figure 4:**
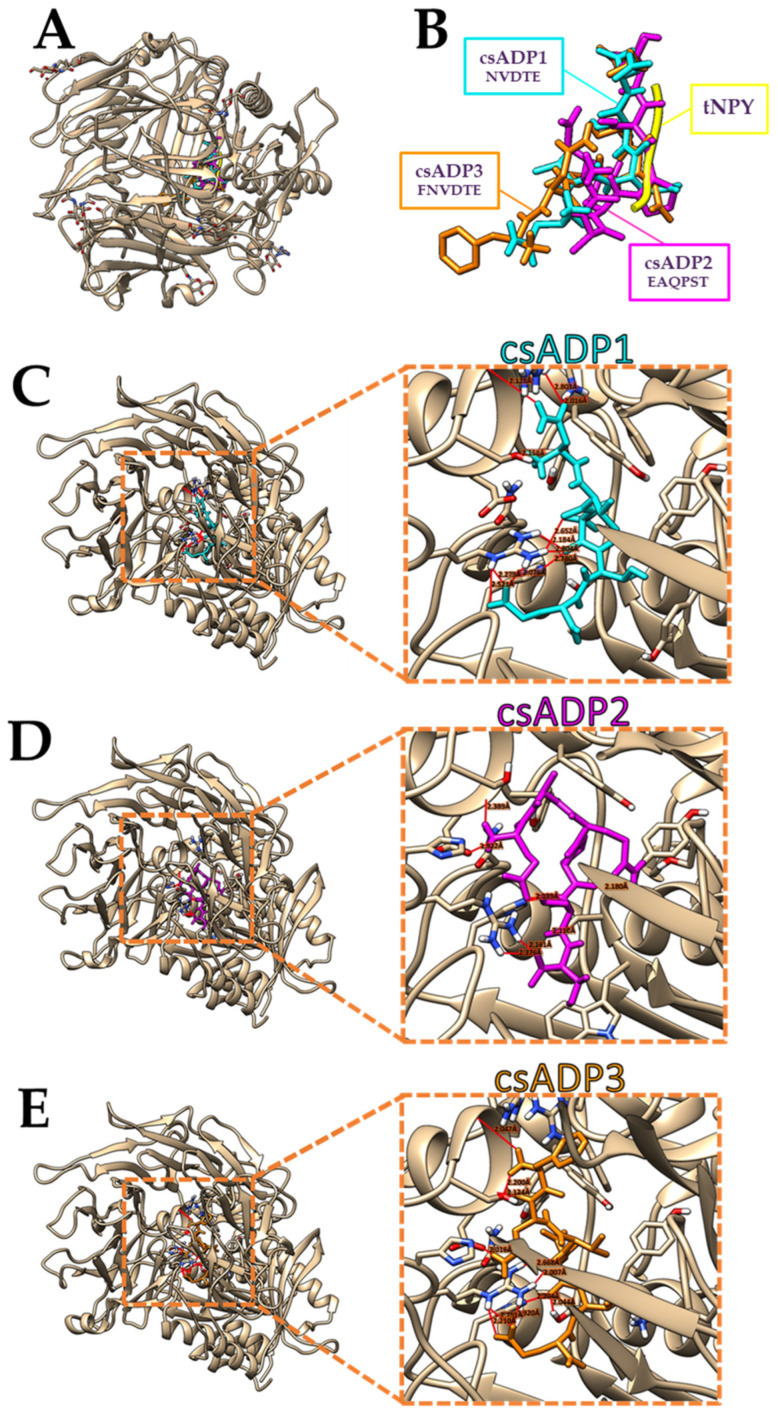
Comparative molecular docking of the three top-ranked *Cannabis sativa* ADPs (csADP1, csADP2, csADP3) and known DPPIV inhibitory peptide (tNPY) on the human crystal structure of DPPIV protease in the apo state (PDB ID: 1R9N) shown with (**A**) and without (**B**) the enzyme structure, and csADP-DPPIV complexes (**C**–**E**). The red lines indicate the hydrogen bonds with the value of bonding distance.

In this procedure, GalaxyPepdock and HPEPDOCK were used to predict the predominant binding modes of a ligand with a protein of known three-dimensional structure. csADP2 and csADP3 were docked with DPPIV protease (PDB ID: 1R9N), which is a crystal structure of human DPPIV imported from the Protein Data Bank. The molecular docking results of these three top-ranked csADPs with a known DPPIV inhibitory peptide (tNPY) from the 1R9N PDB file are shown in Figure 4A,B. All hydrogen bonds observed from the molecular docking of the three peptides (csADP1, csADP2, csADP3) were visualized and calculated using UCSF Chimera, as shown in Figure 4C–E. As a result, most binding sites were located near the active sites of the DPPIV protease structure, as listed in Table 2.

To examine the binding affinity, Prodigy was used to calculate Gibbs free energy and the dissociation constant, as shown in Table 3, along with docking scores from GalaxyPepdock and HPEPDOCK and antidiabetic scores from AntiDMPred. Moreover, intra-chain and inter-chain interactions of various bonds were generated by RING (Table 4). The results of molecular docking simulations demonstrated the existence of hydrogen bond binding near active sites. DPPIV was composed of five subsites including S_1_, S_1_’, S_2_, S_2_’, and S_2_ extensive. The active binding pocket of DPPIV at the N-terminal consisted of catalytic triad Ser630, Asp708, and His740. Also, S_1_’ and S_2_ pockets consisted of protein around active sites. The S_1_ pocket comprised Ser630, Tyr547, Trp659, Tyr631, Asn710, Tyr662, Val656, Tyr666, and Val711, whereas the S_2_ pocket comprised Arg125, Glu205, Glu206, and Pro550 residues. The S_2_ extensive subsite enclosing the S_2_ pocket consisted of Asp708, Trp627, Lys544, Arg358, Val207, Ser209, and Phe357 residues. In addition, Ser630 was located on the nucleophilic elbow, which is essential for DPPIV activity [50]. Compared to other known DPPIV inhibitor drugs (Sitagliptin, Vildagliptin, and Linagliptin), they were reported to bind to the S_1_, S_2_, and S_2_ extensive; S_1_ and S_2_; and S_1_, S_2_, S_1_’, and S_2_’ subsites, respectively [51]. Our results show strong H-bonds between our peptide candidates and several specific residues of the DPPIV active site. The common sites for H-bond formation on the DPPIV active site among these three csADPs were Arg125, Val207, Ser209, and Ser630. Our selected csADPs not only appeared to bind to the subsites of DPPIV inhibitory drugs but also directly binded to the active sites of DPPIV. Moreover, these three csADPs binded near tNPY, the known DPPIV inhibitory peptide included in the 1R9N PDB file [4]. From the energy distribution analysis by the RING program, we found that the three top-ranked csADPs were likely to form Van der Waals and H-bonds with DPPIV. Considering the distances that USCF Chimera computed, csADP1 formed six bonds with distances lower than 2.5 Å, csADP2 formed seven bonds with distances lower than 2.5 Å, and csADP3 formed six bonds with distances lower than 2.5 Å. These H-bonds could be considered strong, mostly covalent bonds [52]. Interestingly, from our results, Arg and Glu seemed to generate strong bonds as well as Glu and Glu between csADP-DPPIV complexes. To select the best two candidates, we primarily considered the docking scores from GalaxyPepDock and HPEPDOCK, as well as the binding affinity indicated by the Gibbs free energy (ΔG) and the dissociation constant (Kd). In terms of ligand binding, ΔG and Kd could be considered as binding energy [53,54]. The lower ΔG and Kd, the greater the binding affinity. According to the programs’ instructions, a lower score indicated better performance for HPEPDOCK, while a higher score was better for GalaxyPepDock. As a result, csADP2 and csADP3 were chosen for further validation by molecular dynamic simulation.

### 2.3. Molecular Dynamics Stimulation

**Figure 5:**
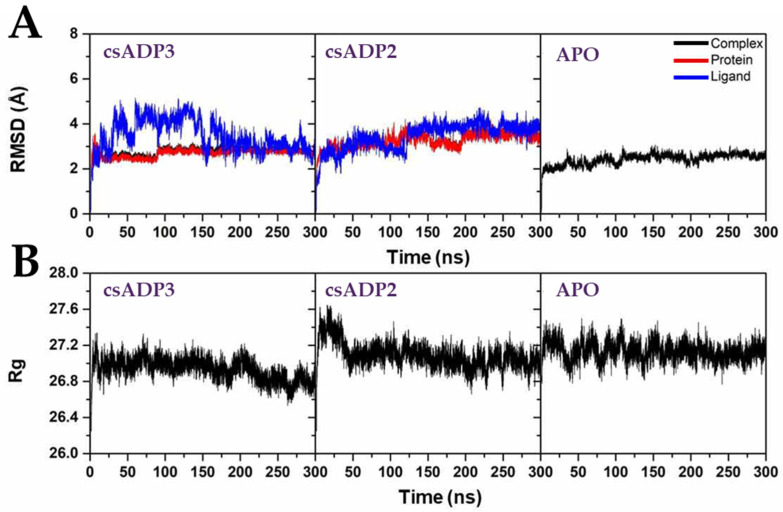
Molecular dynamics simulation of the DPPIV-csADP2 complex, DPPIV-csADP3 complex, and apo state of DPPIV (APO); root mean square deviation (**A**); and radius of gyration (**B**).

**Figure 6:**
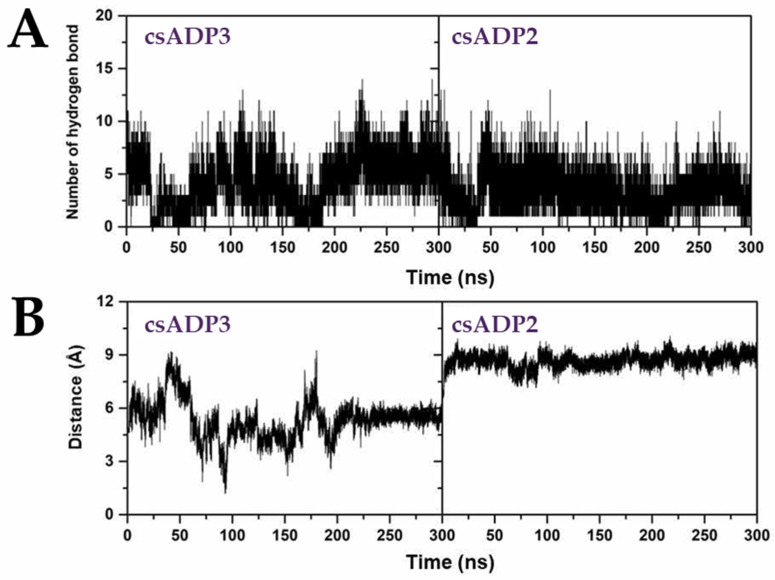
Molecular dynamics simulation of the DPPIV-csADP2 complex and DPPIV-csADP3 complex, number of hydrogen bonds (**A**), and distance (**B**).

Based on the highest-ranked binding affinity and docking scores, csADP2 and csADP3 were selected as candidates for molecular dynamic simulation for 300 nanoseconds to examine the conformational stability and fluctuation analysis of the DPPIV-csADP complexes. To analyze the deviation of the complexes, the root mean square deviation (RMSD) was calculated along with the MD simulation. According to the MD simulation results, the RMSD of complexes and DPPIV significantly overlapped in both csADP2 and csADP3, which indicated the stability of the complex through deviation. As shown in Figure 5A, the deviation of DPPIV (red line) overlapped with the deviation of the complex (red line) in both csADP2 and csADP3. The radius of gyration (Rg) was determined for the structural activity of the macromolecule. The result in Figure 5B shows that the Rg level of csADP3 fluctuated between 26.8 and 27.2 Å, while csADP2 showed a fluctuation between 26.9 and 27.3 Å. The Rg level of ADP 3 fluctuated between 26.8 and 27.2 Å, while csADP2 showed a fluctuation between 26.9 and 27.3 Å. These fluctuations were slightly lower compared to several previous studies [55,56]. Moreover, the number of hydrogen bonds was also considered to examine the dynamic equilibration of the complex system. As a result, the number of hydrogen bonds of csADP3 in the MD simulation was in the range of 3–10 bonds, while that of csADP2 was in the range of 2–7 bonds (Figure 6A). The distance of DPPIV and csADPs shown in Figure 6B demonstrates the strength of the bonds. The results for the number of hydrogen bonds can be interpreted as a continuation of H-bond forming. For csADP3 in the first 155 ns, there were a few hydrogen bonds for some periods, but, after that, it seemed to stabilize at a range of 5 to 10 hydrogen bonds. For csADP2, the formation of hydrogen bonds continued from 75 to 200 ns and from 225 to 375 ns. The distances between the ligands (csADP2 and csADP3) and DPPIV showed stability through their overall slopes. The stability of the csADP2-DPPIV complex appeared to be higher than that of the csADP3-PVVIV complex. However, in terms of DPPIV inhibition, csADP3 might be more efficient due to the shorter distances between the hydrogen bond acceptor–donor pairs at 4.0–6.0 Å and 7.5–9.0 Å, respectively.

Based on recent research, DPPIV appears to be the most promising target for diabetic treatment. Several studies have indicated that serum DPPIV activity is significantly higher in diabetic patients compared to non-diabetic individuals. This heightened expression in serum suggests that targeting DPPIV could play a crucial role in managing and potentially improving diabetic conditions [6,57]. The observed serum DPPIV activity was not attributable to a mutation or structural change in the protein motif. Instead, it reflected the actual concentration of DPPIV enzymes in the patient’s blood. Incretin hormones are gut peptides released into the blood after meal ingestion that stimulate insulin secretion in response to high blood sugar [58]. Since incretin is a substrate of the DPPIV enzyme, a study examining serum DPPIV activity found that blood DPPIV levels were significantly higher in type 2 diabetic Korean patients compared to non-diabetic individuals, resulting in lower incretin levels in the blood [59]. Enhanced insulin secretion occurs more significantly when glucose is ingested orally compared to intravenously, a phenomenon referred to as the incretin effect. Consequently, inhibiting serum DPPIV activity can sustain this effect, leading to reduced blood sugar levels in type 2 diabetes patients.

From our hypothesis, hemp seed peptides might exhibit antidiabetic properties comparable to those of edible plant-derived peptides, as reported in previous studies. For example, short, low-molecular-weight peptides from Bambara bean (hydrolyzed by alcalase); soy, quinoa, and lupine (subjected to sequential hydrolysis with subtilisin–trypsin–flavourzyme); and jack bean (digested by pepsin–pancreatin) have been experimentally shown to possess DPPIV inhibitory properties through in vitro enzyme activity tests [18,29,60,61]. Currently, the idea of predicting and simulating the digestion of plant high-abundance proteins through gastrointestinal tract enzymes (pepsin, trypsin, pancreatin, and chymotrypsin) to represent the peptidome profile in the human gut after a meal has been applied to many edible plants [21,24,29,62,63,64]. This approach allows for the observation of not only the availability of these bioactive peptides but also their absorption potential using the everted gut sac method [29]. Through computer-aided analysis, in silico studies employing bioinformatics approaches have identified potential antidiabetic peptides in macadamia, soybean, black bean, and hemp [17,18,34,36,40,41]. Together with our virtual screening and validation results, these findings suggest that hemp seed peptides could play a therapeutic role in managing diabetes through DPPIV inhibition. However, experimental confirmation of enzyme inhibition is still needed after computer simulations because in silico studies, while useful for predicting potential interactions and identifying promising candidates, cannot fully replicate the complex biological environments in which these peptides will function. Laboratory experiments are essential to validate these predictions, ensuring that the peptides effectively inhibit DPPIV in real biological systems, assessing their stability, bioavailability, and potential side effects and confirming their overall therapeutic efficacy in managing diabetes.

## 3. Materials and Methods

### 3.1. Hemp Seed Putative Hydrolyzed Peptidome Dataset Preparation and Computer-Aided DPPIV Inhibitory Peptide Screening

The workflow began with the major three putative proteins in hemp seed: albumin, edestin, and vicilin (from the National Center for Biotechnology Information: NCBI with specific accession numbers SNQ45452 (albumin), SNQ45159 (edestin1), SNQ45196 (edestin2), SNQ45160 (edestin3), XP_030504501 (vicilin-like seed storage protein At2g28490), XP_030498944 (vicilin-like seed storage protein At2g18540), SNQ45153 (7S vicilin-like protein), and XP_030508281 (vicilin C72-like), respectively), which were then cut into peptides in FASTA format with three digestive enzymes (pepsin, trypsin, and chymotrypsin) by the cleaver R package (version: 4.3.1; [31]). Due to program limitations and typical DPPIV inhibitory peptide lengths [39,46], we examined only 2 to 20 amino acids of peptides. The obtained putative peptidome dataset of a total of 2393 putative hemp seed hydrolyzed peptide sequences was used as input for further sequence-based bioinformatics prediction. iDPPIV-SCM (https://camt.pythonanywhere.com/iDPPIV-SCM, accessed on 21 August 2023) [32] was used to predict the DPPIV inhibitory score value of each peptide. The peptides that passed the criteria (≥294) were classed as DPPIV inhibitory peptides (ADPs) and, to check these ADPs with other properties for certainness, side effects on other cell properties were considered with the following programs: AllerCatPro (https://allercatpro.bii.a-star.edu.sg/, accessed on 28 August 2023) [33] to determine non-allergenicity, HemoPred (http://codes.bio/hemopred/, accessed on 4 September 2023) [34] to determine levels of hemolysis, and ToxinPred2 (https://webs.iiitd.edu.in/raghava/toxinpred2/, accessed on 5 September 2023) [35] to determine toxicity. In addition, the water solubility of peptides was calculated by PepCalc (https://pepcalc.com/, accessed on 11 September 2023) [36] and Peptide Analyzing Tool to Assist Peptide Design (https://www.genscript.com/tools/peptide-analyzing-tool, accessed on 13 September 2023) [37] for further in vitro experiments. For compositional analysis between the significant ADPs and non-ADPs, COPid was employed (http://crdd.osdd.net/raghava/copid/, accessed on 15 September 2023) [38]. Venny 2.1.0 (https://bioinfogp.cnb.csic.es/tools/venny/index.html, accessed on 28 September 2023) [39] was used to generate Venn diagrams for the intersection of all properties analyzed. Finally, 113 peptides were assorted as ADPs and the three top-ranked peptides by iDPPIV-SCM scores were selected for molecular docking simulations.

### 3.2. Protein–Peptide Docking Simulations

The crystal structure of DPPIV in the apo state with PDB ID code 1R9N [4] proteases was accessed from the Protein Data Bank (PDB) (https://www.rcsb.org/structure/1R9N, accessed on 2 September 2023)

The amino acid sequences of each selected csADP candidate (csADP1, csADP2, and csADP3) were docked to the 3D structure of DPPIV protease enzymes using GalaxyPepDock (http://galaxy.seoklab.org/pepdock, accessed on 9 October 2023) [65] and HPEPDOCK (http://huanglab.phys.hust.edu.cn/hpepdock/, accessed on 16 October 2023) [66]. The docking results of the best model and hydrogen bond findings were visualized by the USCF Chimera program (https://www.cgl.ucsf.edu/chimera/, accessed on 26 October 2023) [67]. To calculate the binding affinity, PRODIGY (https://wenmr.science.uu.nl/prodigy, accessed on 30 October 2023) [68] was employed. RING (https://ring.biocomputingup.it/, accessed on 6 November 2023) [69] was used to investigate various bond interactions. In addition, AntiDMPred (http://i.uestc.edu.cn/AntiDMPpred/cgi-bin/AntiDMPpred.pl, accessed on 13 November 2023) [70] was used to predict the desired antidiabetic potentials for consideration.

### 3.3. Molecular Dynamics Simulations

The Amber ff14SB force field and Amber20 software package [71] were employed for MD simulations of three systems. To neutralize the simulated systems, sodium ions were added, and the TIP3P water model was employed to solvate each system, maintaining a distance of 12 Å from the protein (DPPIV protease). For both csADPs and the apo state of DPPIV proteins (APO), the simulation box dimensions were 78 × 76 × 77 Å^3^, 77 × 74 × 77 Å^3^, and 76 × 70 × 86 Å^3^, respectively. The protein–ligand complexes (csADP3, csADP2, and APO) had 734, 734, and 728 residues, respectively. The entire system was solvated at a distance of 12 Å from the protein surface. The system approximate atoms of TIP3P water models for csADP3, csADP2, and APO were 27,333, 26,478, and 28,223 atoms, respectively. The initial conformations were heated to 300 K with a canonical ensemble (NVT) for 100 ps before being equilibrated for another 1200 ps. Then, until 300 ns of the production run, all-atom MD simulations were performed under the isothermal–isobaric ensemble (NPT) at 1 atm and 300 K, with a simulation time step of 2 fs. A Berendsen barostat [72] with a pressure–relaxation time of 1 ps and a Langevin thermostat [73] with a collision frequency of 2 ps^−1^ were used to maintain pressure and temperature during MD simulation, respectively. The SHAKE algorithm [72] constrained all chemical bonds involving hydrogen atoms, while the particle mesh Ewald (PME) summation method [74] handled long-range electrostatic interactions. A cut-off of 10 Å was set for non-bonded interactions. The CPPTRAJ module of AMBER20 was employed to compute specific structural analysis parameters, including root mean square deviation (RMSD), root mean square fluctuation (RMSF), radius of gyration (Rg), and hydrogen bond profile. These parameters were examined over the 300 ns simulation period. To examine the time-dependent properties of all potential protein–ligand interactions, we conducted RING analysis using the RING 3.0 program [69,72]. Various intra-protein interactions were observed in complex systems, including π-π stacking, ionic bonding, hydrogen bonding, and van der Waals interactions. Ten models from the final 100 ns of the 300 ns simulation time were selected for analysis.

## 4. Conclusions

In conclusion, our developed bioinformatics pipeline demonstrated that in silico prediction and validation tools are useful for DPPIV inhibitor screening, offering a time- and cost-effective alternative. Through computer-aided screening of DPPIV inhibitory peptides from the putative peptidome of hemp seeds using our developed bioinformatics pipeline, we revealed two csADP candidates with the most potential, with good predicted binding affinity, high docking scores, and good stability of molecular interactions. According to the predictive results, FNVDTE from edestin and EAQPST from vicilin were identified as promising candidates for DPPIV inhibitors. These findings not only contribute to the exploration of alternative DPPIV inhibitors but also highlight the promise of hemp seed peptides in addressing the needs of individuals with diabetes. Moving forward, further research and experimentation are warranted to fully realize the practical applications of these peptides in functional foods and diabetic management.

## Figures and Tables

**Table 1:** The *Cannabis sativa* antidiabetic peptides (csADPs) with prediction scores from iDPPIV-SCM and non-allergenic, non-hemolytic, and non-toxic results from AllerCatPro, HemoPred, and ToxinPred.

Peptide ID	Sequence (Protein/Protease)	Length	iDPPIV-SCM Prediction Scores	AllerCatPro	HemoPred	ToxinPred
csADP1	NVDTE (Edestin2/Pepsin)	5	335.75	No evidence	Non-hemolytic	0.59
csADP2	EAQPST (Vicilin/Pepsin)	6	325.6	No evidence	Non-hemolytic	0.49
csADP3	FNVDTE (Edestin2/Pepsin)	6	322.6	No evidence	Non-hemolytic	0.57

**Table 2:** List of hydrogen bonds observed from the molecular docking of three peptides (csADP1, csADP2, csADP3) to the human crystal structure of DPPIV protease (PDB ID: 1R9N).

Peptide ID	Secondary Structures	csADP Residues	DPPIV Residues	Distance (Å)
csADP1	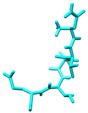	Glu5	Arg125	2.273
		Glu5	Arg125	2.521
		Asp3	Arg125	2.184
		Val2	Arg125	2.652
		Asp3	Arg125	2.604
		Glu5	Arg125	1.976
		Asn1	Arg669	2.803
		Asn1	Arg669	2.016
		Asn1	Ser209	2.158
		Asn1	Val207	2.128
csADP2	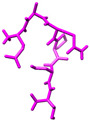	Thr6	Arg125	2.376
		Thr6	Arg125	2.161
		Ser5	Ser630	2.001
		Pro4	Tyr631	2.18
		Glu1	Glu205	2.389
		Glu1	Glu205	1.922
		Ser5	His740	2.152
csADP3	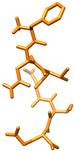	Glu6	Arg125	2.757
		Glu6	Arg125	2.24
		Val3	Arg125	2.668
		Val3	Arg125	2.007
		Glu6	Arg125	1.92
		Asp4	Ser630	2.044
		Phe1	Val207	2.047
		Phe1	Ser630	2.2
		Asn2	Glu205	2.124
		Asn2	Glu205	2.016

**Table 3:** Summary of Gibbs free energy and the dissociation constant (Kd) calculated by PRODIGY, docking scores from GalaxyPepdock and HPEPDOCK, and antidiabetic prediction scores from AntiDMPred.

Peptide ID	PRODIGY		Molecular Docking Scores		AntiDMPred
	ΔG (kcal mol^−1^)	Kd (M) at 25 °C	GalaxyPepDock	HPEPDOCK	
csADP1	−10.2	3.50 × 10^−7^	0.872	−114.142	0.51
csADP2	−11.7	2.50 × 10^−9^	0.954	−143.62	0.50
csADP3	−10.6	1.80 × 10^−8^	0.872	−169.78	0.47

**Table 4:** Summary of intra-chain and inter-chain contacts with a frequency of 50–100% in 10 models found by RING at distance thresholds (Å): H-bond 3.9, Ionic 4, π-cation 5, π-π stacking 6.5, Disulfide 2.5, Van Der Waals 0.01.

Bond	csADP1		csADP2		csADP3	
	Intra	Inter	Intra	Inter	Intra	Inter
H-Bond	573	7	567	7	561	9
π-π Stacking	83	0	80	0	82	1
π-Cation	4	0	3	0	5	0
Ionic	15	1	15	0	11	1
Disulfide	0	0	0	0	0	0
π-H-Bond	2	0	4	1	4	1
Van der Waals	623	7	606	8	622	14

## Data Availability

Data are contained within the article.
